# Thermal Evaluation of Bone Drilling with a One-Drill Protocol

**DOI:** 10.3390/bioengineering11101022

**Published:** 2024-10-14

**Authors:** Sihana Rugova, Marcus Abboud

**Affiliations:** 1 Department of Oral Biology and Pathology, Stony Brook University, Stony Brook, NY 11794, USA; 2 School of Engineering, Stony Brook University, Stony Brook, NY 11794, USA

**Keywords:** bone drilling, infrared thermography, orthopedic surgery, implant dentistry, heat, osteotomy, bone cutting, sequential drilling, pilot drill bit

## Abstract

This study evaluates the thermal impact of a one-drill protocol for osteotomy preparation in dental implant surgery. Our findings demonstrate a significant reduction in heat generation compared to traditional sequential drilling, suggesting potential benefits for implant osseointegration and patient comfort. Specifically, the one-drill protocol was associated with lower peak temperatures and a reduced duration of elevated temperatures. These findings suggest that the one-drill protocol may contribute to improved implant stability and reduce the risk of thermal-induced bone damage. While further research is needed to confirm these findings in clinical settings, the results of this study provide promising evidence for the potential advantages of the one-drill protocol in dental implant surgery. Additionally, the one-drill protocol may offer simplified surgical workflows and reduced instrument management, potentially leading to improved efficiency and cost-effectiveness in dental implant procedures.

## 1. Introduction

As Albrektsson states in his chain for success, “a reliable osseointegration of a bone implant is dependent on the simultaneous control of several parameters such as material biocompatibility, implant design, implant surface, status of the implant bed, surgical technique, and loading conditions [1]”. Each element is integral; failure in any can compromise implant longevity, irrespective of other controls. Dental implants have undergone significant evolution with comprehensive evaluations across these parameters [2]. Titanium remains the preferred biocompatible material, closely followed by zirconia [3,4,5]. Modern designs, particularly tapered endosseous implants with self-tapping threads, are favored for their enhanced primary stability [2,6,7,8]. Research indicates that moderate surface roughness promotes optimal bone response, with ongoing exploration into nano-roughness [9,10,11,12,13]. In terms of loading protocols, delayed implantation and loading are commonly preferred among dentists for predictable healing [5,14]. Surgical techniques vary based on treatment plans and aesthetic considerations, yet they regularly prioritize navigating bone quality and quantity [15,16,17]. While the status of the implant bed traditionally emphasizes bone health, recent studies have also begun exploring the thermal aspects of surgery, examining how heat generation during bone drilling and cutting procedures impact outcomes [18,19,20,21,22,23,24,25,26,27].

In a previous study, we assessed the thermal effects of producing an osteotomy when drilling in the conventional way with a series of drill bits incrementally increasing in size and found that sequential drilling is not sufficient for heat mitigation [28]. In this study, we assess the thermal effects of producing an osteotomy through a one-drill protocol and explore modifications to influence heat generation utilizing an internally developed drill bit. Rather than using conventional drilling for implant bed preparation where drills bits are used in series of incrementally increasing diameters, a single drill bit is used to create the final osteotomy, making that drill bit both the pilot and final drill bit.

## 2. Materials and Methods

**Figure 1:**
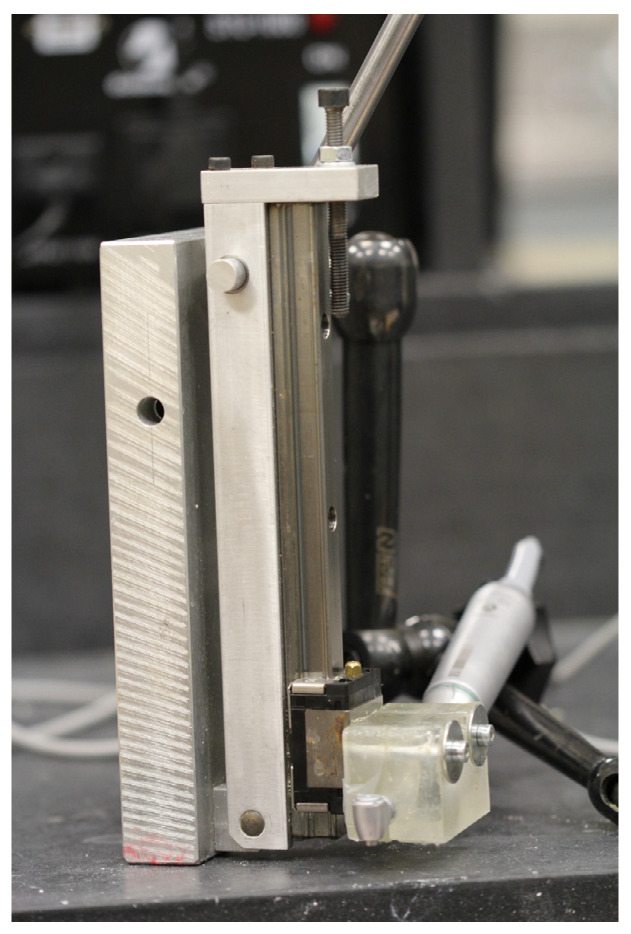
Photo of custom drill press used in this study.

**Figure 2:**
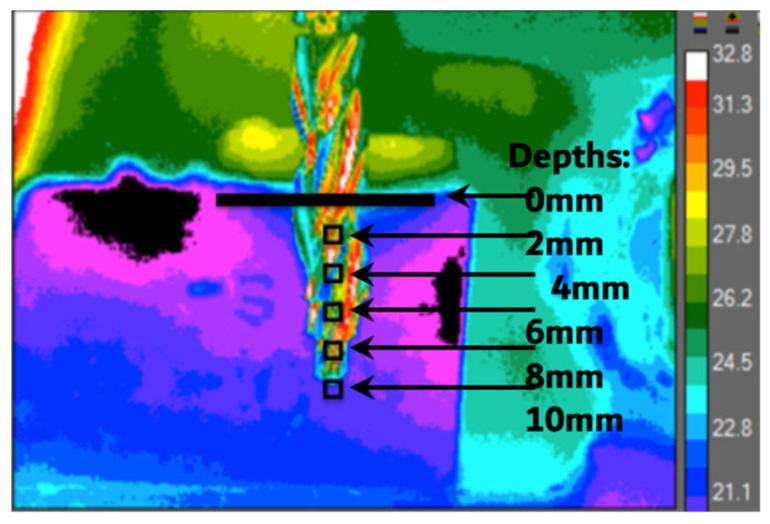
A still image of the ⌀4.1mm-diameter surgical drill bit placed in front of the bone simile taken by the infrared camera.

The testing procedure was carried out according to Rugova and Abboud 2024 [29]. A custom-built drill press with a W&H implant motor (W&H Group, Bürmoos, Austria) was used to ensure a standard, unbiased drilling procedure for each osteotomy (Figure 1). Ten mm-deep osteotomies were drilled into artificial bone similes (BS180035-120035-180035, BoneSim, Cassopolis, MI, USA) to ensure drilling occurred in a standardized bone medium to prevent results from being influenced by inconsistent bone densities. A high-precision Computer Numeric Control (CNC) machine was used both to cut the BoneSims and to mark each osteotomy site. The BoneSim strips were soaked in room temperature saline for 20 min prior to drilling to simulate a more fluid environment. External irrigation at a flow rate of approximately 12 mL/min was used throughout while an infrared camera (FLIR A325sc infrared camera, FLIR Systems Inc., Wilsonville, OR, USA) fixed with a close-up 4× lens acquired temperature data from the surface of the bone 0.5 mm away from the final osteotomy. Figure 2 shows a still image taken with the infrared camera of the largest drill bit used in this study in front of the bone simile in a dynamic rainbow palette.

**Figure 3:**
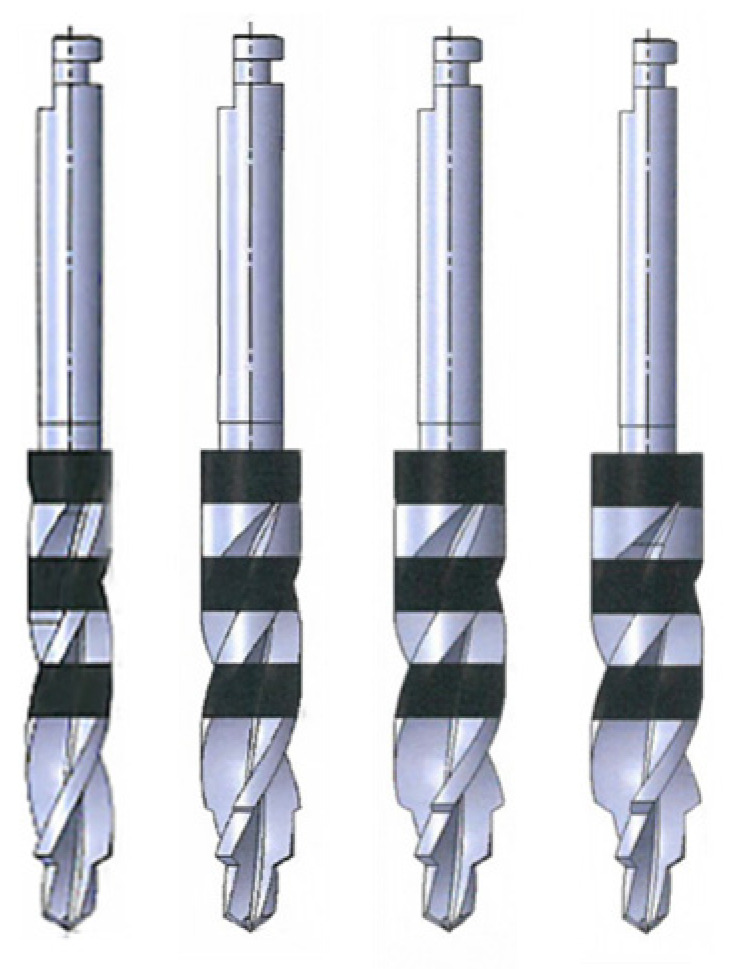
Image of drill bits used in this study. From left to right: ⌀ 3.2 mm, ⌀ 3.3 mm, ⌀ 4.0 mm, and ⌀ 4.1 mm.

Drills and Drilling Groups: Figure 3 shows the four drill bits (Blitz GmbH, Munich, Germany) tested using a load of 2.3 kg and three different spindle speeds (shown in Table 1). The drill bits are meant for a one-drill protocol and, therefore, do not require a ⌀ 2.0 mm drill bit. These drill bits cut primarily at the head/tip rather than its sides. The implant motors used for surgical implant drilling are not used at a higher rpm than 2000.

Videos of the osteotomy procedure were recorded in a dynamic rainbow palette (20 colors) using FLIR ResearchIR Max version 1 on Windows 8.1. Temperature readings were recorded continuously before, during, and after the osteotomy procedure to determine the maximum bone temperatures (Celsius) and duration (seconds) of temperature influence. Base temperatures were normalized to 32 °C to match the maximum maxillary temperature recorded in the literature [17].

Statistical Analysis: The data analysis for this study was generated on Microsoft Excel (Microsoft, Washington, DC, USA, Version 16, 2021). The temperatures achieved under each condition were recorded and compared statistically using the Student’s *t*-test. Statistical significance was established as *p* < 0.05; n = 10.

Tissue damage is both time- and temperature-dependent. The threshold for irreversible tissue damage in this study was 50 °C for 30 s. If the temperature remains in the range of 50–70 °C for 30 s or longer, the damage produced is irreversible. Temperatures at or above 70 °C indicate immediate osteocyte death regardless of duration.

Thermal video recordings were analyzed to document the maximum temperature readings at 5 regions of interest, at depths of 0, 2, 4, 6, 8, and 10 mm, 0.5 mm away from the periphery of the osteotomy throughout the duration of the procedure.

## 3. Results

**Figure 4:**
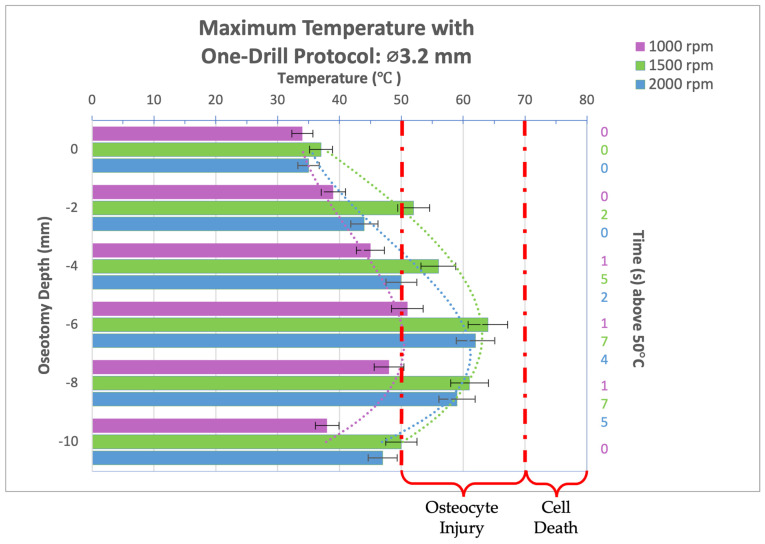
Bar graph showing maximum temperatures reached when drilling into bone to a depth of 10 mm with the 3.2 mm-diameter drill bit at a load of 2.3 kg and three different spindle speeds (1000, 1500, and 2000 rpm). Temperature range of bone cell injury and bone cell death are marked on graph. Left axis shows drilling depth (mm) while top axis shows maximum temperature reached (°C) at that depth. Right axis shows duration (seconds) temperatures exceeded 50 °C.

Thermal video recordings taken during the implant bed preparation procedure for the 3.2 mm-diameter drill bit used in a one-drill protocol were analyzed and maximum temperatures at osteotomy depths of 0, 2, 4, 6, 8, and 10 mm were recorded and organized into the bar graph seen in Figure 4. Compared to an rpm of 1500 or 2000, at 1000 rpm, the 3.2 mm-diameter drill bit produced the lowest maximum temperatures regardless of the osteotomy depth, a significant difference from the 1500 rpm group, which produced the highest maximum temperatures. In the 1000 rpm group, no thermal trauma can be expected as the highest temperature reached was 51 °C for 1 s. All rpms reached their peak temperature at a depth of 6 mm. In all groups, the temperature threshold for irreversible tissue damage, 50 °C for 30 s or 70 °C for 0 s, was not reached. While some reversible cellular injury can be expected at an osteotomy depth of 4, 6, and 8mm for spindle speeds of 1500 and 2000 rpm, the thermal trauma would be biologically mild and insignificant.

**Figure 5:**
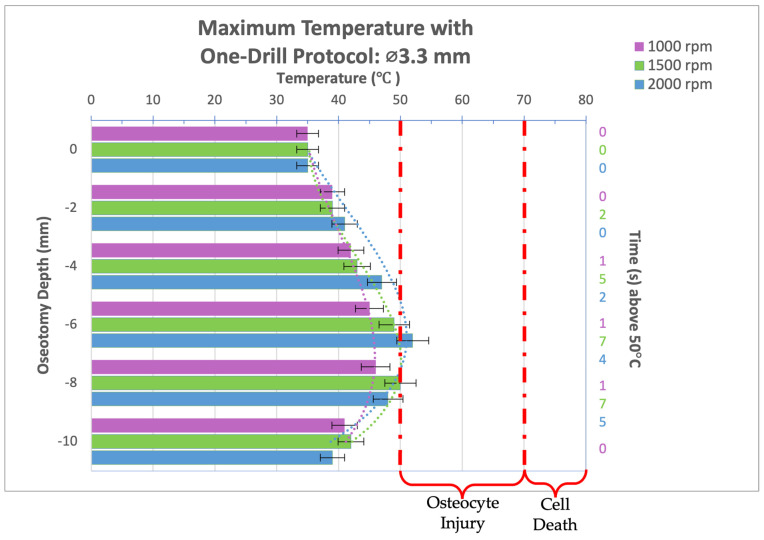
Bar graph showing maximum temperatures reached when drilling into bone to a depth of 10 mm with the 3.3 mm diameter drill bit at a load of 2.3 kg and three different spindle speeds (1000, 1500, and 2000 rpm). Temperature range of bone cell injury and bone cell death are marked on graph. Left axis shows drilling depth (mm) while top axis shows maximum temperature reached (°C) at that depth. Right axis shows duration (seconds) temperatures exceeded 50 °C.

Infrared video recordings taken during the osteotomy procedure for a 3.3 mm-diameter drill bit used in a one-drill protocol were analyzed and maximum temperatures at osteotomy depths of 0, 2, 4, 6, 8, and 10 mm were recorded and organized into the bar graph seen in Figure 5. A depth of 8 mm, and maximum temperatures of 46 °C and 50 °C were reached for 1000 and 1500 rpm, respectively, while 2000 rpm reached a peak temperature of 52 °C at the 6 mm osteotomy depth. There was no significant difference between the three spindle speeds evaluated in all groups. In all groups, the temperature threshold for irreversible tissue damage was not reached. While some reversible cellular injury can be expected at an osteotomy depth of 6 and 8 mm for spindle speeds of 2000 and 1500 rpm, respectively, the thermal trauma would be biologically mild and insignificant.

**Figure 6:**
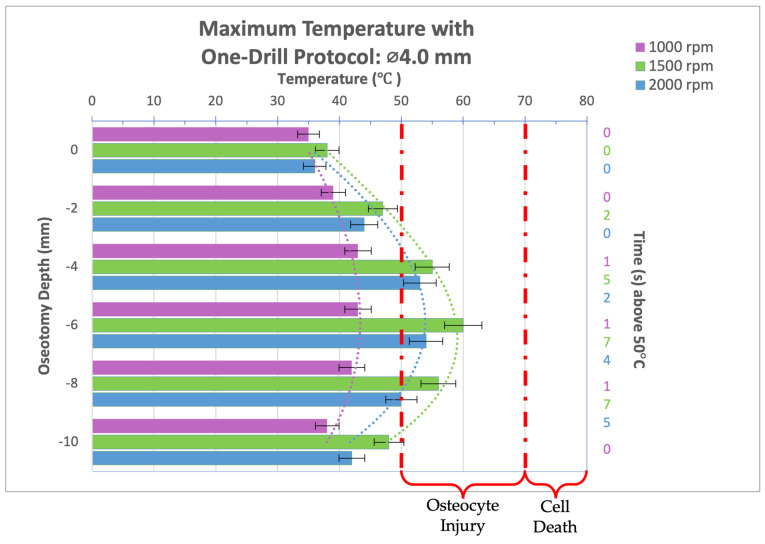
Bar graph showing maximum temperatures reached when drilling into bone to a depth of 10 mm with the 4.0 mm diameter drill bit at a load of 2.3 kg and three different spindle speeds (1000, 1500, and 2000 rpm). Temperature range of bone cell injury and bone cell death are marked on graph. Left axis shows drilling depth (mm) while top axis shows maximum temperature reached (°C) at that depth. Right axis shows duration (seconds) temperatures exceeded 50 °C.

Thermal video recordings taken during the implant bed preparation procedure for a 4.0 mm diameter drill bit used in a one-drill protocol were analyzed and maximum temperatures at osteotomy depths of 0, 2, 4, 6, 8, and 10 mm were recorded and organized into the bar graph seen in Figure 6. Compared to a spindle speed of 1500 or 2000 rpm, at 1000 rpm, the 4.0 mm diameter drill bit produced the lowest maximum temperatures regardless of the osteotomy depth, a significant difference to the 1500 rpm group at osteotomy depths past 2 mm, which produced the highest maximum temperatures. In the 1000 rpm group, no thermal trauma can be expected as temperatures did not exceed 50 °C. All spindle speeds reached a peak temperature at a depth of 6 mm. In all groups, the temperature threshold for irreversible tissue damage, 50 °C for 30 s or 70 °C for 0 s, was not reached. While some reversible cellular injury can be expected at an osteotomy depth of 4, 6, and 8 mm for spindle speeds of 1500 and 2000 rpm, the thermal trauma would be biologically mild and insignificant.

**Figure 7:**
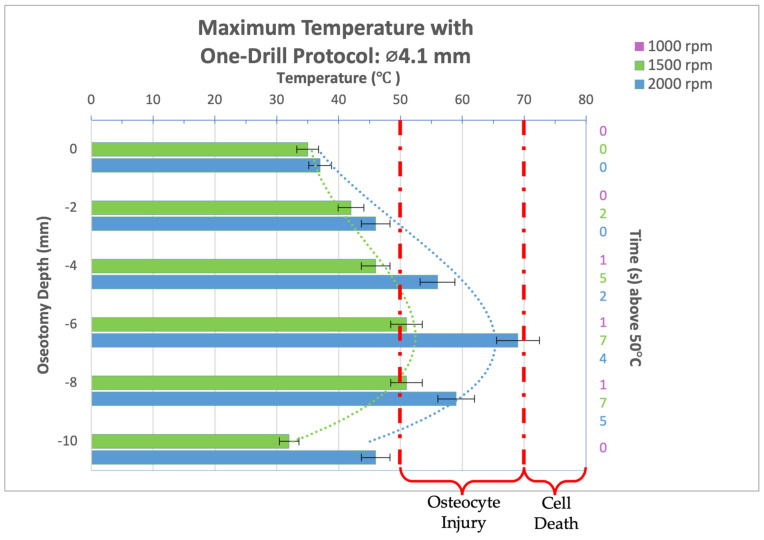
Bar graph showing maximum temperatures reached when drilling into bone to a depth of 10 mm with the 4.1 mm diameter drill bit at a load of 2.3 kg and three different spindle speeds (1000, 1500, and 2000 rpm). Temperature range of bone cell injury and bone cell death are marked on graph. Left axis shows drilling depth (mm) while top axis shows maximum temperature reached (°C) at that depth. Right axis shows duration (seconds) temperatures exceeded 50 °C.

Infrared video recordings taken during the osteotomy procedure for a 4.1 mm diameter drill bit used in a one-drill protocol were analyzed and maximum temperatures at osteotomy depths of 0, 2, 4, 6, 8, and 10 mm were recorded and organized into the bar graph seen in Figure 7. The 1000 rpm group is not shown as this spindle speed was too low for the drill bit to penetrate the cortical portion of the bone present without increasing the load. The 1500 rpm group reached 51 °C for 1 and 2 s at an osteotomy depth of 6 and 8 mm, respectively; however, the reversible cellular injury expected is biologically mild and insignificant. The peak temperatures reached at 2000 rpm also indicate reversible cellular injury limited to the immediate surrounding bone of the implant bed at a depth of 4, 6, and 8 mm. At a depth of 6 mm at 2000 rpm, 69 °C is reached just brushing the threshold for immediate cell death.

## 4. Discussion

Sequential drilling has long been the standard practice in dental implantology, based on the assumption that larger diameter drill bits would remove tissue damage caused by smaller diameter predecessors. However, recent research by Rugova and Abboud 2024 challenges this notion [28]. Those findings indicate that sequential drilling can exacerbate thermal trauma produced by the first drill bit which often exceeds temperatures of 140 °C. The results of this study indicate sequential drilling is not necessary, especially when using drill bits engineered to mitigate thermal trauma, as a one-drill approach can successfully prepare the implant site within a safe temperature range. Other studies have also found a reduced drilling protocol to be beneficial.

Koutiech et. al. (2022) [30] compared conventional sequential drilling to a single-drill protocol and found that the latter generated less heat. This difference was attributed to the drill designs used. The one-drill protocol employed tri-fluted drill bits with a larger flute volume, allowing for better evacuation of bone chips. In contrast, the gradual twist drill bits used in sequence had two flutes and a “nonworking tip design”, which increased the drill bit-to-bone contact area and heat generation [30]. Bettach et al. (2015) also found a one-drill protocol allowed for a faster surgical procedure, increased patient comfort during surgery, and decreased post-surgical discomfort requiring less prescription pain management [31].

Minimally invasive surgical treatments are always preferred due to their reduced trauma and faster healing times. A single-drill protocol is a less invasive approach requiring fewer instruments and a shorter procedure, preserving the healing potential of surrounding tissues, improving osseointegration of the implant, and accelerating the time to final restoration. Supplementary Video S1 demonstrates the temperatures achieved during drilling with a 4.0 mm diameter drill bit in a one-drill protocol at a spindle speed of 1000 rpm. The osteotomy takes about 10 s, and the temperatures reached are below the threshold of damage. In fact, the heat produced that continues to spread after the osteotomy is completed is so low that this temperature range can be considered stimulating for bone [32]. These benefits are particularly important in full-arch implant treatments, immediate loading cases, and for patients with complex medical histories or wound healing challenges. During treatment, patients can experience a more comfortable procedure due to its shortened length and reduced vibrations in bone from the osteotomy, a sensation that cannot be alleviated by anesthesia. Post-surgical, patients can expect a decreased need for pain management and faster recovery times [31].

While bone drill bits designed to mitigate heat can be beneficial in a single drill protocol, such a protocol is not strictly necessary [26]. The effectiveness of a drill bit often depends on the surgeon’s experience and preferences. A one-drill protocol may be more suitable for surgeons with greater experience or who use surgical guides, as it offers less flexibility for correcting misalignments. Those who prefer sequential drilling for greater control of the osteotomy axis or the ability to undersize an osteotomy can also use these instruments. Regardless of the protocol used, it is always recommended to use new drill bits for each patient to reduce the risk of utilizing dull instruments that can increase tissue trauma. Dull drill bits are associated with increased heat generation, especially in cortical bone where a sharp instrument is needed to break through the bone quickly to avoid increased friction and possible excessive loading forces that can cause a clinician to lose operative control [33,34].

For staff, a one-drill or reduced drilling protocol can streamline inventory management and instrument maintenance. By reducing the number of drill bits used, staff can more easily track inventory and schedule replacements. Additionally, a single larger drill bit can harvest a greater amount of bone during the osteotomy, simplifying the process for surgical assistants. This efficiency can contribute to a smoother surgical workflow and reduce overall procedure time.

According to the data in this study, a drill bit with three steps generates less heat compared to a similar design with only two steps, primarily because it distributes the cutting force more effectively and reduces friction during the drilling process. The reduction in friction directly leads to less heat production. One contributing factor to this reduced friction is the gradual removal of material: with three steps, the drill bit removes material in smaller increments at each stage. This incremental material removal lowers the cutting load on each step, requiring less force to drill through the material. Consequently, the gentler removal of material results in lower heat generation from friction.

The presence of an extra step further decreases friction at each stage. With an additional step, each cutting edge engages with the material over a shorter distance, thereby reducing the contact area and friction at each step. The smaller cutting steps also allow for better heat dissipation between each stage. The time between cutting actions provides the drill bit and the material a brief opportunity to cool before the next step engages.

This study demonstrates that a higher spindle speed during bone drilling typically results in elevated temperatures due to several interrelated factors. As the drill bit rotates faster, the frequency of contact between its cutting edges and the bone increases, generating greater friction and, consequently, more heat. Additionally, the rapid rotation associated with higher spindle speed leaves little time for the bone and drill bit to cool between each cutting action. This continuous, high-speed contact causes heat to accumulate quickly, raising the overall temperature.

At high spindle speeds, the drill bit may also struggle to efficiently remove bone debris, such as chips and dust, from the drilling site. When this debris clogs the flutes of the drill bit, it further increases friction and heat, as the bit grinds against both the bone and the trapped debris. Moreover, the mechanical energy from the drill’s motor is transferred to the bone more rapidly at higher spindle speeds. This swift energy transfer, coupled with the increased friction, leads to a significant rise in temperature.

Bone, a viscoelastic material, responds to stress depending on the rate at which it is applied. Under rapid drilling conditions, the bone undergoes more significant deformation, which can increase the amount of heat generated due to internal friction within the bone itself. These combined factors cause a substantial increase in temperature, which can be detrimental during surgical procedures, as overheating the bone may lead to tissue damage and other complications.

The findings of this study underscore the importance for manufacturers to determine the optimal spindle speed for each drill bit used in clinical bone drilling procedures, to minimize the risk of overheating and ensure patient safety.

## 5. Conclusions

The results of this study demonstrate the feasibility and benefits of a one-drill protocol for osteotomy preparation in dental implant surgery. While further research is needed to fully elucidate its long-term clinical implications, the findings suggest that this approach can effectively reduce thermal damage and potentially improve implant outcomes. The ability to achieve a well-prepared osteotomy with a single drill bit offers advantages in terms of efficiency, reduced trauma, and simplified surgical workflow. As dental implant technology continues to evolve, the one-drill protocol or a workflow with a reduced number of drill bits may play a significant role in enhancing patient care and improving the overall success of implant procedures. Based on the results of this study, it can be summarized that a three-step drill bit design generates less heat resulting in a cooler and more efficient drilling process compared to a two-step drill bit. By distributing the cutting force across three steps, the overall force required at each stage is reduced. This lower force diminishes the amount of energy converted into heat, leading to a cooler operation, and likely stimulating bone. The study results should alert clinicians to the potential increase in heat generated by using higher spindle speeds during bone drilling.

## Figures and Tables

**Table 1:** Table showing drill bits tested and the spindle speeds used for each drill bit.

Drill Bits with One-Drill Protocol	Spindle Speeds Tested (rpm)
⌀3.2 mm -1st diameter: 2.0 mm -2nd diameter: 3.2 mm	1000
	1500
	2000
⌀3.3 mm -1st diameter: 2.0 mm -2nd diameter: 3.2 mm -3rd diameter: 3.3 mm	1000
	1500
	2000
⌀4.0 mm -1st diameter: 2.0 mm -2nd diameter: 3.2 mm -3rd diameter: 4.0 mm	1000
	1500
	2000
⌀4.1 mm -1st diameter: 2.5 mm -2nd diameter: 4.0 mm -3rd diameter: 4.1 mm	1000
	1500
	2000

## Data Availability

Dataset available on request from the authors.

## Supplementary Materials

The following supporting information can be downloaded at: https://www.mdpi.com/article/10.3390/bioengineering11101022/s1, Video S1: One-Drill Osteotomy with 4.0mm diameter drill bit.
